# Ten-year risk assessment for cardiovascular diseases using ASCVD risk estimator plus: outcomes from hypertension and diabetes patients

**DOI:** 10.1186/s13098-023-01170-2

**Published:** 2023-10-27

**Authors:** Ian Osoro, Ranjeet Kumar, Amit Sharma

**Affiliations:** Department of Pharmacy Practice, ISF College of Pharmacy, Moga, Punjab 142001 India

**Keywords:** Cardiovascular disease, Indians, Risk prediction model, ASCVD risk estimator plus, Hypertension, Diabetes

## Abstract

**Background:**

Cardiovascular risk prediction models encompass numerous CVD risk factors. Available prediction models were developed from non-Asian cohorts hence we decided to evaluate the performance of the ASCVD risk estimator model and the associated 10-year CVD predisposing factors in Punjab.

**Methods:**

We carried out a cross-sectional study among patients having hypertension and diabetes mellitus in a tertiary hospital in Punjab, India. 201 participants without ASCVD who were ≥ 40 years old and had been admitted to the medical ward were assessed. a pre-validated questionnaire was used to collect data on the socio-demographics and behavioral patterns. Lipid profile and blood pressure measurements were collected as per standard protocols. The respondents’ CVD risk was assessed using ASCVD Risk Estimator Plus. Data were analyzed using IBM SPSS version 26; bivariate analysis was done using Chi-square and binary logistic regression was used to identify the predictors of 10-year risk for CVD at a 5% level of significance.

Measurements.

We examined the stratification of the predicted outcomes and evaluated the associations between individual risk factors and the predicted cardiovascular events. Our study categorized the results of these outcomes into 4 categories: low category (1–5%), borderline category (6–9%) intermediate category (10–20%), and high category (21–95%).

**Results:**

Out of the 201 participants that enrolled in our study, the majority 76 (37.8%) were in the intermediate category, 56 (27.9%) were in the high category, 41 (20.4%) were in the borderline category, 28 (13.9%) were in the low category. The median ASCVD percentage was 14.20%. Respondents who were alcoholics, smokers, and drug abusers (OR = 5.8, 95% CI 0.397–83.584) were associated with the highest likelihood of developing CVDs. Furthermore, males had a significantly higher mean predicted CVD outcome % (M = 23.18%) compared to females (M = 14.91%).

**Conclusion:**

According to our prediction study, it was discovered that 145 (72.1%) participants were not likely to have had an ASCVD in the next 10 years. However, middle-aged males should be more cautious with their lifestyle habits, particularly in dealing with risk factors that can expose them to CVDs.

## Introduction

According to the World Health Organization (WHO), about 17.9 million deaths reported globally in 2019 resulted from cardiovascular diseases. These accounted for 32% of the total deaths making them the leading cause of deaths globally. Stroke and heart failure accounted for 85% of these deaths. Notably, these conditions can be prevented by addressing behavioral risk factors such as tobacco use, unhealthy diet and obesity, physical inactivity, and harmful use of alcohol [1]. According to a study that evaluated the changing patterns in India from 1990 to 2016, it was discovered that CVD diseases contributed to 28.1% of the total deaths. These deaths also increased from 1.3 million in 1990 to 2.8 million in 2016 with Kerala and Punjab having the leading prevalence rates [2].

The current epidemiological evidence suggests an elevated percentage of CVD diseases among Indians. This is a source of concern considering the incidence rate of these diseases is higher in the younger age. About 291 million people had CVDs in 1990 however this number has extremely skyrocketed to 523 million in 2019. Alternatively, the number of CVD deaths rose from 12.1 million in 1990 to 18.6 million in 2019 globally [3]. The catastrophic effects of CVDs continue to be experienced globally and particularly in low-income countries where lifestyle, and poor healthcare among other factors are prevalent. A cardiovascular risk calculator is a screening tool that is used in cardiovascular risk assessment. These tools mainly predict the risk of an individual developing a heart condition in the future (i.e., 5 years, 10 years, or lifetime. The concept of cardiovascular risk assessment was introduced by the Framingham heart study in 1948 in the USA. It was the first cardiovascular observational longitudinal cohort study established to identify the epidemiology and risk factors for cardiovascular diseases.

The early death of USA President Franklin Delano Roosevelt in 1945 led to the development of the National Heart Institute currently named the National Heart, Lung, and Blood Institute in 1948. This study was meant to be a 20-year study however by 1968, the study had yet to accomplish its objectives and hence it was extended through funds from donors along with a supplementary grant from President Richard Nixon. Kannel, Truett, and Cornfield published the first multivariable risk function for atherosclerotic heart disease in 1967. In 1976, Kannel and colleagues established the first risk profile having a general cardiovascular occurrence as the endpoint. In 1998, the Framingham Risk Score for coronary heart disease was developed by Wilson and colleagues and it was the best risk profile until that time. Subsequently, it became the base risk profile for the development of other cardiovascular risk profiles [4].

## Need for cardiovascular risk assessment

According to WHO, cardiovascular diseases are the leading cause of death worldwide (17.9 million) as well as in India (272 per 100,000 persons) [5]. Indians are at a higher risk of developing cardiovascular diseases than their Western counterparts and it has been reported by WHO that one-fifth of the global CVD deaths arise from India. Some of the major concerns of CVD condition in Indians are the early age onset, the rapid deterioration, and increasing mortality rates [6]. It is important to detect cardiovascular disease as early as possible so that management with counseling and medication can begin. Cardiovascular risk prediction models play a crucial role in the prevention and management of the CVDs. Various CVD prediction models have been developed worldwide with the Framingham risk score model being the most commonly used [7–9].

Cardiovascular risk prediction models are important in early CVD detection, patient counseling, specific disease surveillance, identifying risks and regional differences, and developing health promotion programs [10]

## Methods

### Study setting

The study was conducted among hospitalized HTN and DM patients in Guru Gobind Singh Medical College and Hospital, Faridkot, Punjab state, India.

### Study design and population

The study was a prospective observational study that included participants of Indian descent only conducted between October 2022 and March 2023. The study population included all the hospitalized HTN and DM patients at the time of study. Inclusion criteria for this study were (≥ 40 years old) patients who had no history of confirmed atherosclerotic cardiovascular disease (ASCVD) at the time of enrolment. Exclusion criteria were based on: the presence of known heart disease (previous MI), history of stroke, and missing laboratory values. The participants were included after giving their informed consent. A total of 201 participants from Punjab state, India enrolled in our study. STROBE guidelines were followed in the data collection.

### Sample size and sampling technique

The sample size was calculated by assuming a 95% confidence interval or 5% level of significance and margin of error as per the literature. The sample size was calculated through the software "epi info" designed by CDC, U.S. Department of Health and Human Services. The expected frequency of the disease was taken to be 50%.

### Study variables

Variables included for calculation of the ASCVD Risk Estimator Plus include gender, age, race, SBP, DBP, anti-hypertensive treatment, smoking, total cholesterol (mg/dL), High-density lipoprotein (HDL), Low-density lipoprotein (LDL), and history of diabetes.

## O*utcome(S)*

The ASCVD Risk Estimator Plus categorizes the predicted 10-year ASCVD risk into: Low-risk (< 5%), Borderline risk (5–7.4%), Intermediate risk (7.5–19.9%), and High risk (≥ 20%). Therefore, our main outcome of interest was the patients categorized as a high-risk group who are likely to develop CVD within the next 10 years.

### Cardiovascular risk prediction models

In our study, the ASCVD Risk Estimator Plus risk prediction model was used to predict 10-year cardiovascular outcomes. The ASCVD Risk Estimator Plus model was developed to minimize the ASCVD overprediction in certain groups of individuals noted in the PCE model. Although it has been noted to overpredict ASCVD outcomes in Asians, it still has been shown to have significant clinical usefulness. Rifai et al. [11] aimed to compare the ASCVD score and coronary artery calcium (CAC) of various races. According to the study, South Asians at low and intermediate levels of ASCVD score were likely to have a zero CAC score. Logistic regression models were used to were used to assess the relationship between ethnicity and ASCVD score. The authors concluded that ASCVCD is likely to overestimate the South Asians who have been categorized as either low or intermediate risk however it can be used for clinical decision making [11].

### Statistical analysis

IBM SPSS Statistics; Software version: 26.

In our study, four different types of statistical tests were applied: Linear regression analysis, chi-square tests, Mann Whitney U test, and Kruskal Wallis analysis using the Statistical Package for Social Sciences (SPSS) Software. For continuous variables, summaries were made using mean, medians, and quartiles, and compared using the Wilcoxon test. Additionally, for categorical variables, summaries were made using frequencies and percentages, and comparison was conducted through McNemar's chi-square test. A p-value < 0.05 was considered significant. For every participant, the predicted likelihood of developing a CVD (i.e., MI, CHD, or stroke) event within the next 10 years was computed using the ASCVD Risk Estimator Plus.

## Results

The baseline characteristics of the study population are presented in Table 1. According to the 2017 new ACC/AHA High Blood Pressure Guidelines Lower Definition of Hypertension, the majority of the study population was in stage 1 hypertension (i.e., Systolic range 130–139 and Diastolic range 80–89). The total cholesterol and LDL were high in a majority of the study population, while the HDL was low.

**Table 1:** CVD prediction variables and their frequencies

Variables	Frequency	Percentage (%)
Smoking status		
Yes	33	16.42
No	168	83.58
Alcohol consumption status		
Alcohol alone	38	18.9
Alcohol + drug abuse	36	17.9
Alcohol + smoking	24	11.9
Alcoholic, smoker, and drug abuse	4	2.0
Drug abuse alone	20	10.0
Not alcoholic, smoker, and drug abuse	79	39.3
Systolic blood pressure		
Normal levels	3	1.5
Elevated levels	199	98.5
Total Cholesterol		
Normal	4	2.0
High	198	98
Plasma HDLc		
Optimum	29	14.43
Low	172	85.57
DM and HTN status		
Only DM	92	45.8
Only HTN	44	21.9
Both DM and HTN	64	31.8
None	1	0.5

*SBP* Systolic Blood Pressure, *DBP* Diastolic Blood Pressure, *TC* Total Cholesterol, *HDL* High-density lipoprotein, *LDL* Low-density lipoprotein

### Socio-demographic characteristics of participants

The mean age of the respondents was 50.4 ± 9.8 years. The minimum age noted in this study was 40 years and the maximum age was 86 years. The majority of the participants came under the age category between 41 and 50 years that is 92 (45.8%) participants. All participants were of Indian descent and the females were slightly more than males i.e., 51.7%.

### Disposition of risk factors among participants

The prevalence of drinking alcohol alone, alcohol and drug abuse, alcohol and smoking, combined alcohol, smoking, and drug abuse, and engaging in drug abuse alone was found to be (18.9%, 17.9%, 11.9%, 2.0%, 10.0%) respectively. Systolic hypertension, hyperlipidemia, low HDLc, high LDLc, diabetes, and HTN presence had a prevalence of 198 (98.5%), 197 (98.0%), 154 (76.6%), 177 (84.6%), and 200 (99.5%) respectively as seen in Table 1 below.

### Stratification of CVD risk among hospitalized participants

**Fig. 1:**
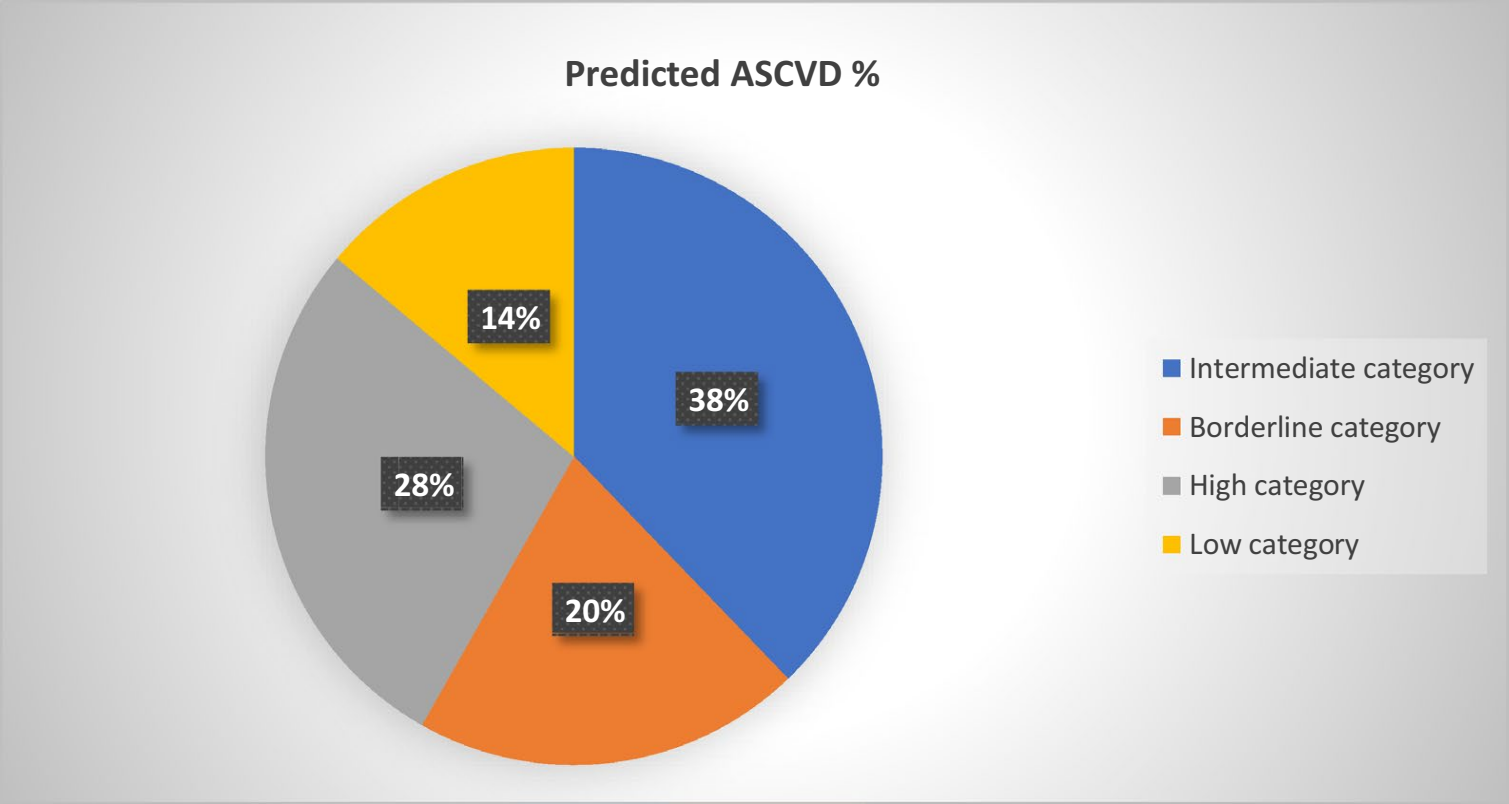
Predicted ASCVD outcomes chart

Out of the 201 participants, the majority 76 (37.8%) were in the intermediate category i.e.,56 (27.9%) were in the high category i.e., 41 (20.4%) were in the borderline category i.e., and 28 (13.9%) were in the low category. The mean ASCVD percentage was 18.94% as seen in Fig. 1 below.

### Association between socio-demographic variables and 10-year risk for CVDs among the respondents

Males had a significantly higher mean predicted CVD outcome % (M = 23.18%) compared to females (M = 14.91%). Females were significantly higher in the not alcoholic, non-smoker, and not a drug abuser (51.9%) category compared to their social habits (*P* = 0.586*)*. A correlation test of Age vs. ASCVD outcomes was conducted with linear regression and the results were found significant as *p* = 0.001, showing age was positively correlated with the predicted CVD %. Table 2 below gives a summary of the statistical tests applied in this study.

**Table 2:** CVD prediction variables and their frequencies

Tests (variables)	*p-value*
Chi-square	
TC cat vs. SBP cat	0.001*
Habits vs Gender	*0.586*
Mann Whitney U Test	
SBP vs Gender	*0.431*
TC vs Gender	*0.372*
ASCVD vs Gender	0.001*
Linear regression	
ASCVD vs Age	0.001*
ASCVD vs TC	0.001*
TC vs Age	*0.382*
Kruskal Wallis	
ASCVD vs. SBP cat	0.027*
ASCVD vs DM history	0.001*
ASCVD vs Habits	0.001*
DM history vs. Age	*0.623*
SBP vs DM History	*0.001*

^*^-Significant

### Association between risk factors and 10-year risk for CVDs among the respondents

The chi-square test revealed that the participant's SBP category was significantly associated with the TC category *(p* = 0.001*).* Additionally, the patients’ habits were not significantly influenced by their gender (*p* = 0.586*).* The Mann Whitney u test revealed that gender did not affect SBP and TC respectively (*p* = 0.431*, p* = 0.372). Linear regression analysis showed that age influenced the ASCVD outcomes (*p* = 0.001) and TC levels were associated with the predicted ASCVD outcomes *(p* = 0.001). however, the patient's age did not influence their TC levels. Kruskal Wallis tests showed that: SBP category did not affect predicted ASCVD outcomes (*p* = 0.027), DM history and habits are significantly associated with ASCVD outcomes (*p* = 0.001*, *0.001 respectively), age does not influence DM history *(p* = 0.623*)* and finally, DM history significantly influence the SBP outcome.

## Discussion

Participants who were only drug abusers were about 2 times associated with a greater probability of developing CVD compared to those who were sober/ did not abuse drugs. Some of the drugs being abused were charas and bhang and it is known that the consumption of cannabis products imposes a risk of tachycardia.

Respondents who combined smoking and alcoholism were 5 times associated with a higher likelihood of developing CVD compared to those who were sober. Participants who combined alcoholism and drug abuse were about 4 times linked with a higher probability of developing CVD compared to those who were sober. Respondents who are alcoholics, smokers, and drug abusers are about 6 times more likely to develop CVD as compared to their sober counterparts as seen in Table 3 below. Gender was discovered to be one of the factors of 10- a year risk of CVDs. Participants who were males were about 4 times associated with a higher likelihood of developing CVD compared to females. It is known that males are at a higher susceptibility to developing CVD in comparison to females.

**Table 3:** Associated 10-year risk for CVD predictors

variable	Odds ratio	95% confidence interval		*P *value
		Lower	Upper	
Age	0.688	0.601	0.788	0.001*
Habits				
Habits 1	2.116	0.137	32.756	*0.592*
Habits 2	5.251	0.395	69.841	*0.209*
Habits 3	0.244	0.016	3.769	*0.313*
Habits 4	3.896	0.275	55.135	*0.315*
Habits 5	5.762	0.397	83.584	*0.199*
Gender	4.265	1.879	9.683	0.001*

Coding: Habit 1: Drug abuse alone, Habits 2: alcoholic and smoker, Habits 3: Not alcoholic, smoker and drug abuse, Habits 4: alcoholic and drug abuse, Habits 5: alcoholic, smoker and drug abuse

^*^Significant

In our prediction study, the majority of the patients come under the age category between 41 and 50 years that is 92 (45.8%) patients followed by 58 (28.9%) patients who were found in the age group 51–60 years. The mean age was found to be 50.42 years with a minimum age of 40 years and maximum age of 86 years. This reveals that middle-aged adults are increasingly more likely to suffer from CVDs than expected. The same results were reported by [12] who discovered in their study that young to middle-aged adults (50 ± 6 years) were likely to develop CVD conditions due to traditional as well as lifestyle factors. According to their study, factors such as financial stress, higher psychological stress, and habits such as not eating fruits and vegetables at a young age increased the probability of having CVDs in the future. [12]. We have reported significant results when comparing the age category with the patient's social habits. Our study's mean age was compared to the mean age observed with values previously measured at 45 years by [13].

This study also reveals that male patients are more prone to develop CVD events than their female counterparts. We noted that the mean ASCVD score of female patients was statistically significantly lower (M = 14.91%) as compared to the male patients (M = 23.18%). Additionally, our study confirmed that there was no statistically significant difference in TC levels between males and females i.e. mean TC level of female patients (M = 255.42 mg/dL) was just a little lesser compared to male patients (M = 259.67 mg/dL). Our study reported that (51.7%) of the CVD-admitted patients were female, which corresponds with a review by (Gao et al. 2019) which showed that women have a lesser incidence of CVDs than men however they have a higher mortality and more serious prognosis of CVD occurrences [14].

In our study, most patients (38.8%) fall under the SBP range between 133 and 142 mmHg followed by 60 (29.9%) patients found in the SBP group 143–152 mmHg, and the mean SBP was 138.71 mmHg compared to the mean SBP that was observed with values previously measured 122 mmHg [15]. This reveals that the majority of the patients in the medicine wards have their SBP in normal ranges. Moreover, we compared our results to a study by [16] whose SBP measure was 146 mmHg [16]. Our study results indicate that the majority of the patients come under the DBP category between 83 and 92 mmHg 108 (53.7%) followed by 39 (19.4%) patients found in the DBP group 73–82 mmHg. With a mean DBP of 86.7 mmHg, our study participants had a slightly higher DBP than the normal value i.e. 80 mmHg. Furthermore, our DBP results were compared to a study whose observed value was 75 mmHg by [17]. A non-significant association was found between the patients' DBP and their habits using Kruskal Wallis analysis.

During this study, it was found that the majority of the participants came under the TC category between 246 and 265 mg/dL that is 81 (40.3%) patients followed by 54 (26.9%) patients who were found in TC group 266–285 mg/dL. The Median TC was found to be 252.00 mg/dL with a minimum TC of 189 mg/dL and a maximum TC of 320 mg/dL. Our TC results were compared to a study whose observed value was 243 mg/dL by [18].

The study results reveal that the majority of the patients come under the HDL category between 29 and 33 mg/dL that is 58 (28.9%) patients followed by 48 (23.9%) patients who were found in HDL group 19–23 mg/dL. The Median HDL level was found to be 29.00 mg/dL, with a minimum HDL of 20 mg/dL and a maximum TC of 44 mg/dL. In a website article reviewed by Rohra, HDL level < 40 mg/dL is categorized as low level [19]. Therefore, we can infer that from this study, the majority of the patients admitted to the medicine ward were having a lesser HDL level than normal. We compared our HDL results to a study whose observed value was 54 mg/dL [20].

This study results reveal that the majority of the patients came under the LDL category between 186 and 195 mg/dL that is 86 (42.8%) patients followed by 65 (32.3%) patients who were found in LDL group 176–185 mg/dL. Our median LDL was found to be 182.00 mg/dL. With the optimum LDL range being < 100 mg/dL, our results reveal that the majority of the patients admitted to the medicine ward have significantly higher LDL levels than recommended. We compared our study with one conducted by whose mean LDL was 110 mg/dL [21].

Furthermore, we assessed the diabetes and HTN status of our patients and the majority had only DM 92 (45.8%) followed by Only HTN 44 (21.9%). In a study by (Jayaraj et al. 2020), it was noted that the prevalence of diabetes mellitus in patients randomly selected from medicine wards was 32.44% whereas that for hypertension was found to be 38.8%. our study reveals that there is a high prevalence of both HTN and DM in India hence preventive measures ought to be properly addressed when treating the patients [22].

In addition, the majority of our participants were neither not alcoholics, smokers nor drug abusers. However, the remainder of the patients engaged in either alcoholism, smoking, or drug abuse. In a study by [23], the prevalence of alcohol abuse and tobacco consumption in India was discovered to be 8.7% and 7.9% respectively [23]. Our study therefore reveals that in India, age does not significantly influence the social habits of an individual.

The major findings of this study reveal that the majority of the patients 76 (37.8%) were in the intermediate category i.e., 10–20%, followed by 56 (27.9%) patients who were in the high category i.e. 21–95%. Our median ASCVD percentage was 14.20% and importantly, we reveal that 145 (72.1%) patients in our study are not likely to have had an ASCVD in the next 10 years.

## Conclusion

The present prediction study concludes that middle-aged adults should be more aware of their exposure to risks that can enhance CVD development such as bad lifestyle habits. Moreover, we urge responsible bodies such as the government to pay more attention to this age group which is increasingly becoming susceptible to CVD development. Additionally, it is necessary for healthcare practitioners to carefully assess hospitalized patients' blood pressure characteristics and adequately educate them on the best practices to maintain their pressure levels within the required limits. Importantly, the patients are to intentionally monitor their blood pressure levels, eating habits, smoking habits, exercise habits, etc.

Commendably, the Government of India is moving in the right direction in tackling this problem after launching the National Programme for Prevention and Control of Cancer, Diabetes, Cardiovascular Diseases and Stroke (NPCDCS). Soon, India should aim to develop a better CVD prediction model based on their cohorts.

## Data Availability

Upon request, external researchers can be offered access to the data analyzed at the Department of Pharmacy Practice, ISF College of Pharmacy, Moga, Punjab. To do so, kindly contact Dr. Amit Sharma.

## Abbreviation

HTN: Hypertension
DM: Diabetes Mellitus
CVD: Cardiovascular diseases
LDLc: Low-density lipoprotein cholesterol
HDLc: High-density lipoprotein cholesterol

## References

[1] https://www.who.int/news-room/fact-sheets/detail/cardiovascular-diseases-(cvds). Accessed on 15 Mar 2023.

[2] Prabhakaran D, Jeemon P, Sharma M (2018). India State-Level Disease Burden Initiative CVD Collaborators The changing patterns of cardiovascular diseases and their risk factors in the states of India: the Global Burden of Disease Study 1990–2016. Lancet Glob Health.

[3] Roth GA, Mensah GA, Johnson CO (2019). Global burden of cardiovascular diseases and risk factors, 1990–2019: update from the GBD. Study.

[4] Mahmood SS, Levy D, Vasan RS, Wang TJ (2014). The framingham heart study and the epidemiology of cardiovascular disease: a historical perspective. Lancet.

[5] Kundu J, Kundu S (2022). Cardiovascular disease (CVD) and its associated risk factors among older adults in India: evidence from LASI Wave 1. Clinical Epidemiol Global Health.

[6] Sreeniwas Kumar A, Sinha N (2020). Cardiovascular disease in India: A 360-degree overview. Med J Armed Forces India.

[7] Damen JA, Pajouheshnia R, Heus P (2019). Performance of the Framingham risk models and pooled cohort equations for predicting 10-year risk of cardiovascular disease: a systematic review and meta-analysis. BMC Med.

[8] Yang L, Wu H, Jin X (2020). Study of a cardiovascular disease prediction model based on random forest in eastern China. Sci Rep.

[9] Damen JA, Hooft L, Schuit E, Debray TP, Collins GS, Tzoulaki I, Lassale CM, Siontis GC, Chiocchia V, Roberts C, Schlüssel MM, Gerry S, Black JA, Heus P, van der Schouw YT, Peelen LM, Moons KG (2016). Prediction models for cardiovascular disease risk in the general population: systematic review. BMJ.

[10] Qian X, Li Y, Zhang X, Guo H, He J, Wang X, Yan Y, Ma J, Ma R, Guo S (2022). A cardiovascular disease prediction model based on routine physical examination indicators using machine learning methods: a cohort study. Front Cardiovasc Med.

[11] Al Rifai M, Cainzos-Achirica M, Kanaya AM, Kandula NR, Dardardi Z, Joshi PH, Patel J, Budoff M, Yeboah J, Guallar E, Blumenthal RS, Blaha MJ (2018). Discordance between 10-year cardiovascular risk estimates using the ACC/AHA 2013 estimator and coronary artery calcium in individuals from 5 racial/ethnic groups: comparing MASALA and MESA. Atherosclerosis.

[12] Garshick MS, Vaidean GD, Vani A, Underberg JA, Newman JD, Berger JS, Fisher EA, Gianos E (2019). Cardiovascular risk factor control and lifestyle factors in young to middle-aged adults with newly diagnosed obstructive coronary artery disease. Cardiology.

[13] Lloyd-Jones DM, Leip EP, Larson MG, D'Agostino RB, Beiser A, Wilson PW, Wolf PA, Levy D (2006). Prediction of lifetime risk for cardiovascular disease by risk factor burden at 50 years of age. Circulation.

[14] Gao Z, Chen Z, Sun A, Dung X (2019). Gender differences in cardiovascular disease. Med Nov Technol Devices.

[15] Bundy JD, Li C, Stuchlik P, Bu X, Kelly TN, Mills KT, He H, Chen J, Whelton PK, He J (2017). Systolic blood pressure reduction and risk of cardiovascular disease and mortality: a systematic review and network meta-analysis. JAMA Cardiol.

[16] Rahimi et al. (2021) Blood Pressure Lowering Treatment Trialists' Collaboration. Pharmacological blood pressure lowering for primary and secondary prevention of cardiovascular disease across different levels of blood pressure: an individual participant-level data meta-analysis. *Lancet*. 2021 May 1;397(10285):1625–1636. doi: 10.1016/S0140-6736(21)00590-0. Erratum in: *Lancet.* 2021 May 22;397(10288):1884. PMID: 33933205; PMCID: PMC8102467. 10.1016/S0140-6736(21)00590-0 PMC8102467 33933205

[17] Li J, Somers VK, Gao X, Chen Z, Ju J, Lin Q, Mohamed EA, Karim S, Xu H, Zhang L (2021). Evaluation of optimal diastolic blood pressure range among adults with treated systolic blood pressure less than 130 mm Hg. JAMA Netw Open.

[18] Yi SW, Yi JJ, Ohrr H (2019). Total cholesterol and all-cause mortality by sex and age: a prospective cohort study among 128 million adults. Sci Rep.

[19] Rohra D: Cholesterol Levels by Age In India: Normal LDL, HDL, Total Cholesterol Levels in Adults and Teenagers. https://redcliffelabs.com/myhealth/lab-test/heart-test/cholesterol-levels-by-age-in-indiawhat-are-the-total-cholesterol-levels-in-adults-and-teenagers-in-india/. Accessed 14 Mar 2023.

[20] Cho KH (2022). The current status of research on high-density lipoproteins (HDL): a paradigm shift from HDL quantity to HDL quality and HDL functionality. Int J Mol Sci.

[21] Ueda P, Gulayin P, Danaei G (2018). Long-term moderately elevated LDL-cholesterol and blood pressure and risk of coronary heart disease. PLoS ONE.

[22] Jayaraj NP, Shanmugam J, Duraisamy S, Padmavathy L (2020). Prevalence and determinants of hypertension and diabetes mellitus in an urban area of Coimbatore. Intern J Commun Med Public Health.

[23] Sivapuram MS, Nagarathna R, Anand A, Patil S, Singh A, Nagendra HR (2020). Prevalence of alcohol and tobacco use in india and implications for COVID-19—niyantrita madhumeha bharata study projections. J Med Life.

